# Patterns of anxiety and distress over 12 months following participation in HPV primary screening

**DOI:** 10.1136/sextrans-2020-054780

**Published:** 2021-08-03

**Authors:** Laura A V Marlow, Emily McBride, Deborah Ridout, Alice S Forster, Henry Kitchener, Jo Waller

**Affiliations:** 1 Cancer Prevention Group, School of Cancer and Pharmaceutical Sciences, King's College London, London, UK; 2 Research Department of Behavioural Science and Health, University College London, London, UK; 3 UCL Great Ormond Street Institute of Child Health, University College London, London, UK; 4 Women's Cancer Centre, Institute of Cancer Sciences, The University of Manchester, Manchester, UK

**Keywords:** public health, psychology, women's health services, primary health care, delivery of health care

## Abstract

**Objectives:**

Many countries are now using primary human papillomavirus (HPV) testing for cervical screening, testing for high-risk HPV and using cytology as triage. An HPV-positive result can have an adverse psychological impact, at least in the short term. In this paper, we explore the psychological impact of primary HPV screening over 12 months.

**Methods:**

Women were surveyed soon after receiving their results (n=1133) and 6 (n=762) and 12 months (n=537) later. Primary outcomes were anxiety (Short-Form State Anxiety Inventory-6) and distress (General Health Questionnaire-12). Secondary outcomes included concern, worry about cervical cancer and reassurance. Mixed-effects regression models were used to explore differences at each time point and change over time across four groups according to their baseline result: control (HPV negative/HPV cleared/normal cytology and not tested for HPV); HPV positive with normal cytology; HPV positive with abnormal cytology; and HPV persistent (ie, second consecutive HPV-positive result).

**Results:**

Women who were HPV positive with abnormal cytology had the highest anxiety scores at baseline (mean=42.2, SD: 15.0), but this had declined by 12 months (mean=37.0, SD: 11.7) and was closer to being within the ‘normal’ range (scores between 34 and 36 are considered ‘normal’). This group also had the highest distress at baseline (mean=3.3, SD: 3.8, scores of 3+ indicate case-level distress), but the lowest distress at 12 months (mean=1.9, SD: 3.1). At 6 and 12 months, there were no between-group differences in anxiety or distress for any HPV-positive result group when compared with the control group. The control group were less concerned and more reassured about their result at 6 and 12 months than the HPV-positive with normal cytology group.

**Conclusions:**

Our findings suggest the initial adverse impact of an HPV-positive screening result on anxiety and distress diminishes over time. Specific concerns about the result may be longer lasting and efforts should be made to address them.

## Introduction

Tests for human papillomavirus (HPV) DNA are more sensitive than cytology-based screening for the detection of precancerous cervical lesions.^1^ Accordingly, cervical screening is changing from cytology to primary HPV testing.^2^ This involves testing samples for high-risk (hr)HPV and using cytology as triage. Screening programmes that use HPV-based screening are expected to reduce cancer incidence and health system costs.^3^ However, the specificity of HPV-based screening is lower than cytology-based screening and as such will result in more unnecessary referrals.^1^ Women who test hrHPV positive with normal cytology will therefore be invited for early recall 12 months later rather than being referred immediately to colposcopy (see algorithm for HPV primary screening^4^).

In the English HPV primary screening pilot,^5^ 12.7% of women tested positive for hrHPV. Just 4.2% were referred immediately (because of abnormal cytology) and 2.8% (with normal cytology) were later referred at 12-month or 24-month recall. The remaining women who attended recall at 12 or 24 months were no longer HPV positive and returned to standard recall without a recommendation for further investigation. In most cases, testing positive for hrHPV does not have health-related consequences, however it has been associated with elevated anxiety, as well as specific concerns about cancer risk and sexual relationships.^6–13^ Shortly after receiving screening results, women who tested HPV positive for the first time were significantly more anxious than women testing HPV negative or those not tested for HPV, regardless of cytology result.^11^ Concern about results and worry about cervical cancer were also elevated in HPV-positive women, including those receiving a second positive result (indicating persistent infection).

The implications of adverse psychological outcomes will depend in part on how long-lasting they are. Previous studies have suggested that anxiety about HPV results wanes over time. Evaluation of HPV testing for triage (following abnormal cytology) found baseline differences in anxiety and distress were no longer evident at 6 months.^14^ However, concern about results and cancer worry persisted in women with abnormal cytology or HPV-positive results. In Norway, no differences in anxiety and depression were found between women screened with primary HPV-based versus cytology-based algorithms up to 24 months later.^10^ Understanding the longer term impact of early recall, following an HPV-positive/cytology normal result, is important for implementation.

In this paper, we extend our previous report of the short-term psychological impact of receiving different hrHPV and cytology results,^11 15^ repeating the psychological measures at 6-month and 12-month follow-up. Based on previous findings,^14^ and our own baseline results,^11^ we expected that between-group differences in psychological well-being would not be maintained at the follow-up time points but those differences in screening-specific concerns might persist.

## Methods

### Design

Embedded within the HPV primary screening pilot study in England,^5^ we carried out the ‘Psychological Impact of Primary Screening’ (PIPS) Study. PIPS invited a subset of 5494 women to complete a questionnaire shortly after receiving their screening result (baseline).^11^ Selection of women was pragmatic. National Health Service (NHS) staff identified potential participants in batches over several months until the target sample size was achieved. Women who returned their consent form at baseline were sent follow-up questionnaires. This paper reports findings from these follow-up time points. A full protocol for this study has been published.^15^

### Participants

Women were 24–65 years old and had participated in cervical screening (2016–2017) in one of five sites that implemented HPV primary screening in 2013. Women were purposively selected to represent different test results received at baseline (see protocol for detail^15^). Test results were determined from clinical records.

### Procedures

Questionnaires were sent to women 6 months and 12 months after their baseline questionnaire (regardless of participation at 6 months). A reminder was mailed 3 weeks later.

### Measures

The primary outcomes in the questionnaire were anxiety and distress. Anxiety was assessed using the Short-Form State Anxiety Inventory-6, a 6-item measure (range 20–80) with normal range considered to be 34–36.^16^ Distress was assessed using the General Health Questionnaire-12, a 12‐item measure (range 0–12) with >3 indicating case-level distress.^17^

Secondary outcomes included worry about cervical cancer and concern/reassurance about screening results, with single items using 5-point Likert scales, recoded into binary outcomes for analyses: higher worry (score >3, moderately/very worried); higher concern (>3, moderately/very concerned); higher reassurance (>2, somewhat/moderately/very reassured) (see^11^ for details).

In routine management for HPV primary screening, women who are hrHPV positive with normal cytology are recalled for repeat screening at 12 months.^4^ These women could expect a recall appointment around the time of the 12-month questionnaire. Clinical data were not available at 12 months, but we assessed self-reported reattendance at early recall and test results. Women were asked ‘Have you been invited for cervical screening again since the start of this study, about a year ago?’ Responses captured whether they had been invited and/or attended. We also asked ‘Can you remember what your screening result was?’ Response options included HPV and cytology results.

Demographic characteristics were assessed at baseline using self-report (marital status, ethnicity, education) and clinical health records (for age, number of previous screens, deprivation using Index of Multiple Deprivation (IMD) quintile for residential postcode) (see table 1 for details).

**Table 1:** Demographic characteristics of women at baseline, 6 months and 12 months (unweighted)

	Baseline	6 months	12 months
Total n	1133	762	537
Age, mean years (SD)	41.2 (11.8)	42.9 (11.7)	43.2 (12.0)
Marital status, n (%)			
Current partner	859 (75.8)	582 (76.4)	420 (78.2)
No partner	253 (22.3)	170 (22.3)	112 (20.9)
Ethnicity, n (%)			
White (British or other)	1016 (89.7)	702 (92.1)	497 (92.6)
Other ethnicity	91 (8.0)	46 (6.0)	33 (6.1)
Prefer not to say	3 (0.3)	1 (0.1)	0 (0)
IMD quintile, n (%)			
1 (most deprived)	172 (15.2)	97 (12.7)	62 (11.5)
2	211 (18.6)	129 (16.9)	90 (16.8)
3	278 (24.5)	193 (25.3)	155 (28.9)
4	195 (17.2)	140 (18.4)	106 (19.7)
5 (least deprived)	193 (17.0)	145 (19.0)	93 (17.3)
Education, n (%)			
Degree or higher	481 (42.5)	344 (45.1)	247 (46.0)
Qualification below degree	538 (47.5)	355 (46.6)	246 (45.8)
No formal qualifications	83 (7.3)	49 (6.4)	36 (6.7)
Previous screens, mean screens (SD)	6.3 (4.9)	7.0 (4.9)	7.0 (4.8)
NHS site, n (%)			
Liverpool	188 (16.6)	125 (16.4)	96 (17.9)
Sheffield	210 (18.5)	137 (18.0)	112 (20.9)
London North West	148 (13.1)	85 (11.2)	76 (14.2)
Norfolk and Norwich	200 (17.7)	136 (17.8)	122 (22.7)
Manchester	387 (34.2)	279 (36.6)	131 (24.4)
Result group, n (%)			
HPV positive, normal	259 (22.9)	175 (23.0)	109 (20.3)
HPV positive, abnormal	172 (15.2)	107 (14.0)	73 (13.6)
HPV persistent*	179 (15.8)	118 (15.5)	89 (16.6)
Control group†	523 (46.2)	362 (47.5)	266 (49.5)
Not tested for HPV	208 (18.4)	136 (17.8)	101 (18.8)
HPV negative	249 (22.0)	184 (24.1)	130 (24.2)
HPV cleared*	66 (5.8)	42 (5.5)	35 (6.5)

% may not add up to 100% due to missing data; <8% missing for any variable.

*Women who were HPV persistent and HPV cleared had tested HPV positive ~1 year earlier and the baseline questionnaire was following their early recall result.

†The control group included women who were not tested for HPV, HPV negative or HPV cleared.

HPV, human papillomavirus; IMD, Index of Multiple Deprivation; NHS, National Health Service.

### Analyses

Analyses were carried out in SPSS version 25^18^ and Stata.^19^ Since there were no differences in any of the outcomes between those not tested for HPV (the original control group^11^) and the two HPV-negative groups (HPV negative and HPV cleared) at baseline, we made an a priori decision to combine these groups for follow-up analyses. Analyses presented here compare four groups: (1) control (including not tested for HPV, HPV negative, HPV cleared); (2) HPV positive with normal cytology; (3) HPV positive with abnormal cytology; (4) HPV persistent.

We compared rates of attrition at both time points by group and NHS site using X^2^ tests. We compared baseline sociodemographic characteristics between responders and non-responders at both time points using t-tests or X^2^ tests. Baseline analysis demonstrated small variations in demographic characteristics between women recruited into the study and non-responders, so we generated and applied population weights, based on age group and IMD quintile within each test result group.^11^ We used data from 955 387 women who attended HPV primary screening within the sites included in our study in 2017–2018 to calculate weights. Population weights were applied to all regression models.

For each primary outcome (anxiety and distress), we fitted a mixed-effects regression model, including participant as the random effect, time point and result group as the fixed effects and accounting for the longitudinal structure of the data. We investigated the interaction between result group and time point factors and prespecified two main contrasts. First, we compared mean differences for each HPV-positive group with the control group at 6 months and 12 months separately. We compared the HPV-positive with normal cytology group with the HPV-positive with abnormal cytology group at both time points. Second, we compared changes in mean scores between baseline and 6 months and between baseline and 12 months, between each HPV-positive group and the control group.

For the binary outcomes (worry, concern and reassurance), we fitted a logistic regression model to explore whether higher worry, higher concern or lower reassurance at baseline was associated with higher odds of an adverse outcome at 6 and 12 months. We fitted separate models for 6-month and 12-month follow-up and included baseline status (higher/lower) and result group as covariates; we also included the interaction between these terms. For each outcome, the interaction was not significant; therefore, only main effects were included. We have presented results at each time point, adjusted for baseline outcome. For worry, we compared the proportion of women with higher worry between each of the HPV-positive groups and the control group. For concern and reassurance, we used the HPV-positive with normal cytology group as the reference group because of the skewed distribution of responses with very few women concerned or not reassured in the control group.

All models were adjusted for seven prespecified covariates: age, marital status, ethnicity, IMD, education, number of previous screens and NHS site. Full models including all prespecified covariates were fitted, as recommend by Harrell.^20^ We used multiple imputation assuming data were missing at random. The imputation model included primary outcomes and all sociodemographic factors, which we assumed included all predictors of missingness. The final models were derived by fitting appropriate regression models including all covariates, and estimates were combined using Rubin’s rules.^21^

We ran supplementary analyses with the 12-month questionnaire data exploring whether self-reported recall status (reattended, HPV positive; reattended, HPV negative; not yet reattended) was associated with raised anxiety or distress among women who were HPV positive with normal cytology or HPV persistent at their baseline screen. We used t-tests for continuous measures and X^2^ tests for categorical data. Women in the control group were not eligible for early recall.

## Results

### Sample

Baseline questionnaires were returned by 1154 of 5494 women, of whom 21 were ineligible (online supplemental figure S1). Of the 1133 eligible for inclusion and mailed follow-up questionnaires, 67% returned a 6-month (n=762) and 47% returned a 12-month questionnaire (n=537). Only 40% returned questionnaires at all time points (online supplemental table S1). There were no significant differences in participation at 6 months and 12 months by result groups; however, women who participated at follow-up were more likely to be older, from less deprived areas, more highly educated and from a white ethnic background compared with women who did not return the follow-up questionnaires (table 1).

10.1136/sextrans-2020-054780.supp1 Supplementary data

### Anxiety and distress at 6 and 12 months

**Figure 1:**
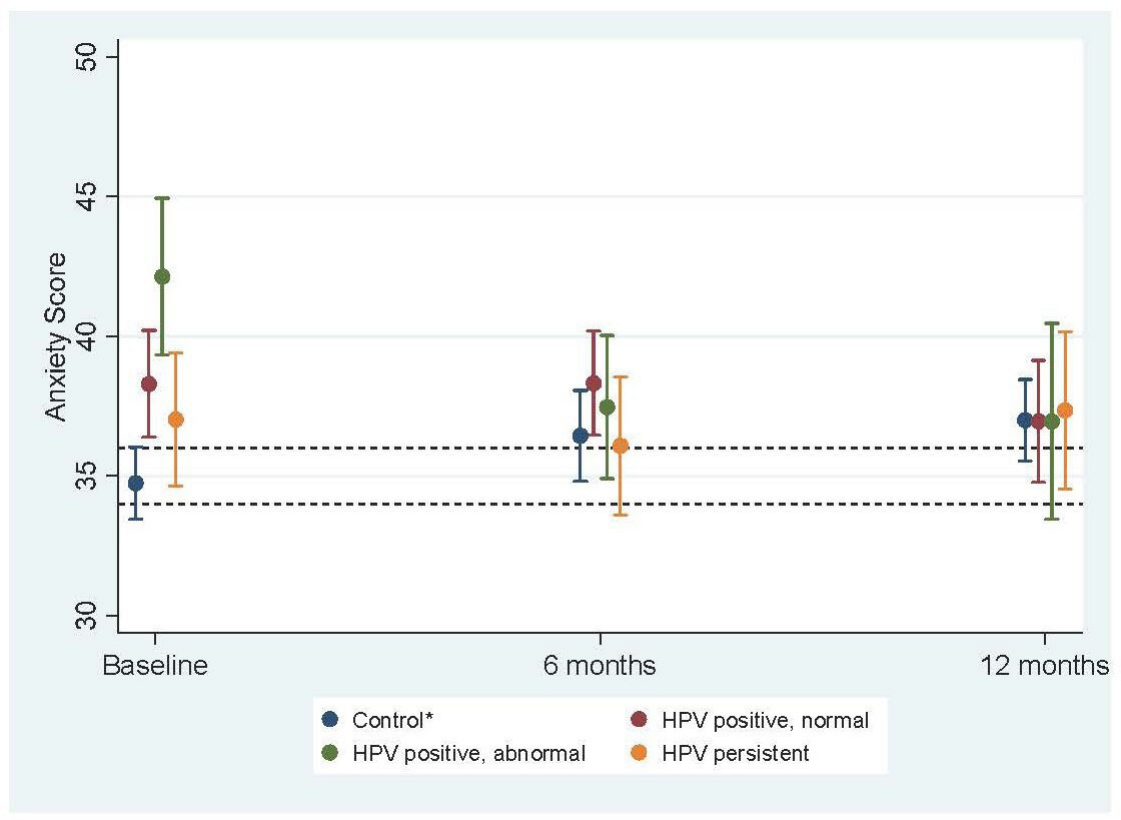
Mean anxiety scores (with 95% CIs) at baseline, 6 months and 12 months across test result groups^a^. *Includes women not tested for HPV, HPV negative, HPV cleared. a: weighted by age group and IMD quintile. Fully adjusted for age, marital status, ethnicity, IMD, education, number of previous screens and NHS site. Note: dotted lines indicate ‘normal’ range, between 34 and 36. HPV, human papillomavirus; IMD, Index of Multiple Deprivation; NHS, National Health Service.

**Figure 2:**
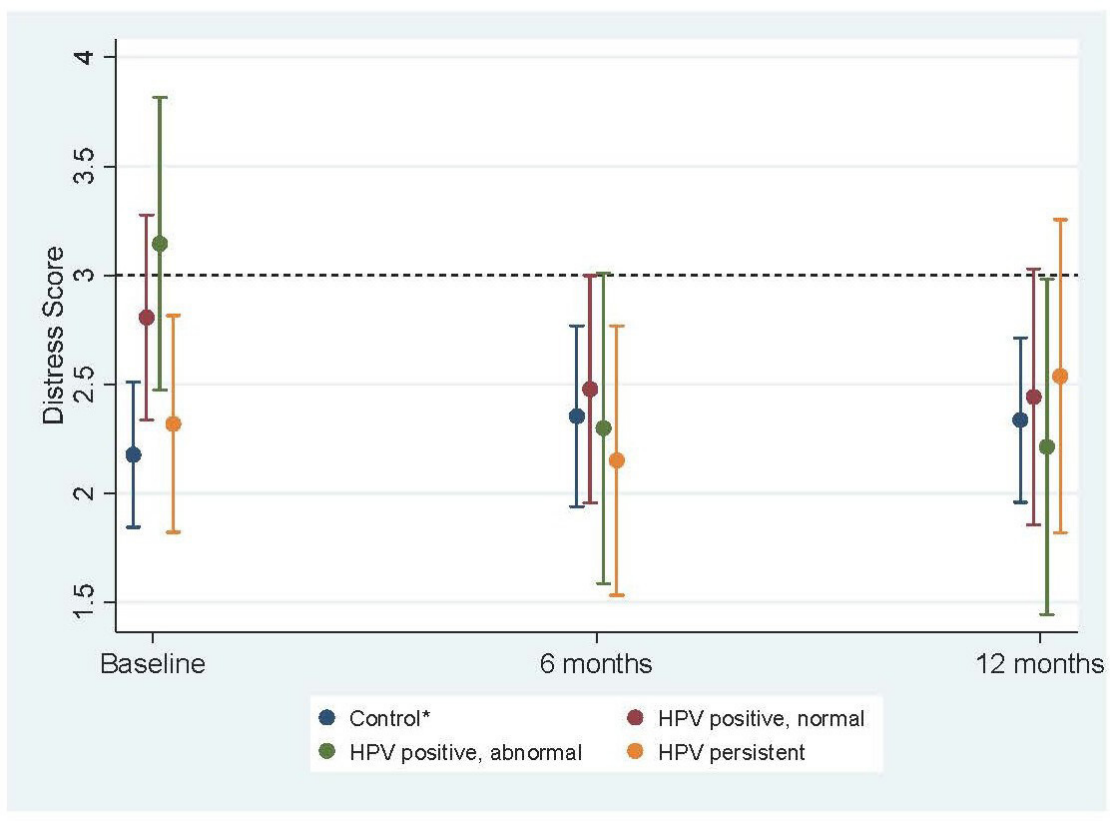
Mean distress scores (with 95% CIs) at baseline, 6 months and 12 months across test result groups^a^. *Includes women not tested for HPV, HPV negative, HPV cleared. a: weighted by age group and IMD quintile. Fully adjusted for age, marital status, ethnicity, IMD, education, number of previous screens and NHS site. Note: dotted line indicates threshold for case-level distress. HPV, human papillomavirus; IMD, Index of Multiple Deprivation; NHS, National Health Service.

At 6-month and 12-month follow-up, there were no significant differences between the control group and any of the HPV-positive groups for anxiety or distress (table 2). For all three HPV-positive groups, the pattern of anxiety and distress scores showed a downward trend between baseline and both follow-up time points (figures 1 and 2). This decrease was greatest and significantly different from change in the control group for the women who were HPV positive with abnormal cytology. Over 12 months, anxiety scores among women in the HPV-positive abnormal cytology group reduced on average by 7.4 more units (95% CI: 2.8 to 12.1) relative to change in the control group (online supplemental table S2). Change in anxiety score was also significantly different for the HPV-positive normal cytology group compared with the control group. Over 12 months, anxiety scores among women in the HPV-positive normal cytology group reduced on average by 3.6 more units (95% CI: 0.2 to 7.0) relative to change in the control group (online supplemental table S2). Change in distress for the HPV-positive groups was significantly different from change in control group for women who were HPV positive with abnormal cytology. Over 12 months, distress scores among women in the HPV-positive abnormal cytology group reduced on average by 1.1 more units (95% CI: 0.1 to 2.1) relative to change in the control group (online supplemental table S2).

**Table 2:** Anxiety and distress at 6 months and 12 months by baseline screening result group

	Control*	HPV positive, normal		HPV positive, abnormal		HPV persistent	
	M (SD)†	M (SD)†	MD (95% CI)‡	M (SD)†	MD (95% CI)‡	M (SD)†	MD (95% CI)‡
STAI score (mean; SD)							
Baseline (n=1009)	34.2 (12.3)	38.3 (14.3)	NR	42.2 (15.0)	NR	36.8 (13.1)	NR
6 months (n=694)	36.1 (12.4)	38.6 (12.3)	1.88 (−0.7 to 4.5)	38.9 (12.7)	1.03 (−2.1 to 4.2)	35.1 (12.0)	−0.4 (−3.2 to 2.4)
12 months (n=493)	37.0 (12.1)	36.0 (13.6)	−0.04 (−2.8 to 2.7)	37.0 (11.7)	−0.1 (−4.0 to 3.9)	36.6 (11.8)	0.3 (−3.0 to 3.7)
GHQ score (mean; SD)							
Baseline (n=1118)	2.1 (3.2)	2.8 (3.6)	NR	3.3 (3.8)	NR	2.45 (3.2)	NR
6 months (n=756)	2.4 (3.6)	2.4 (3.3)	0.12 (−0.58 to 0.83)	2.6 (3.5)	−0.06 (−0.87 to 0.76)	2.43 (3.6)	−0.20 (−0.94 to 0.53)
12 months (n=535)	2.2 (3.0)	2.1 (3.3)	0.11 (−0.57 to 0.78)	1.9 (3.1)	−0.12 (−0.96 to 0.72)	2.40 (3.1)	0.20 (−0.61 to 1.01)

NR means not reported in this paper, see baseline analyses.^10^

*Includes women not tested for HPV, HPV negative, HPV cleared.

†Observed mean (M) and SD.

‡Mean difference (MD) and 95% CI compared with the control group using mixed-effects regression models, weighted and fully adjusted for age, marital status, ethnicity, Index of Multiple Deprivation, education, number of previous screens and NHS site.

GHQ, General Health Questionnaire; HPV, human papillomavirus; NHS, National Health Service; STAI, State Anxiety Inventory.

### Worry about developing cervical cancer

**Figure 3:**
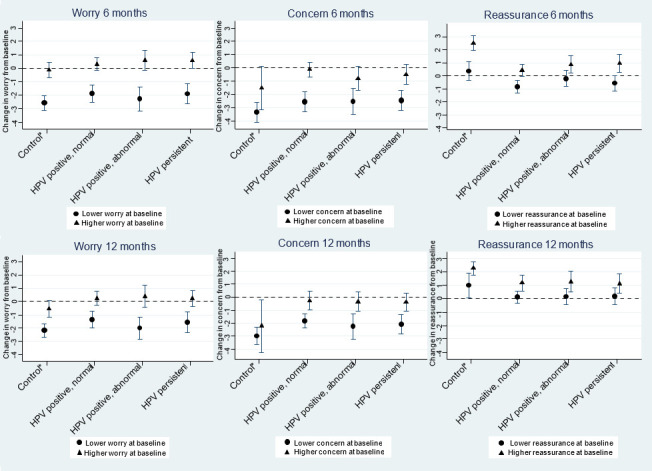
ORs (with 95% CIs) for higher worry about cancer, higher concern and higher reassurance at 6 and 12 months, stratified by whether women were classified as having lower/higher responses at baseline^a^. *Includes women not tested for HPV, HPV negative, HPV cleared. a: weighted by age group and IMD quintile. Fully adjusted for age, marital status, ethnicity, IMD, education, number of previous screens and NHS site. HPV, human papillomavirus; IMD, Index of Multiple Deprivation; NHS, National Health Service.

HPV-persistent women were more likely to be worried about developing cervical cancer than the control group at 6 and 12 months. Women who were HPV positive with normal cytology were more likely to be worried than the control group at 12 months only (table 3). Figure 3 shows the odds of higher worry about cancer for women who were and were not worried at baseline. Across all result groups, women who were worried at baseline were more likely to be worried at 6 and 12 months (OR=11.7, 95% CI: 7.3 to 18.6 and OR=6.1, 95% CI: 3.7 to 9.9, respectively), compared with those not worried at baseline. There was no significant difference in this trend between the result groups at either 6 or 12 months (p=0.4 and p=0.3, respectively).

**Table 3:** Worry, concern and reassurance at 6 months and 12 months*

	6-month follow-up			12-month follow-up		
	Higher worry (n=757)	Higher concern (n=758)	Higher reassurance (n=756)	Higher worry (n=533)	Higher concern (n=533)	Higher reassurance (n=529)
Control†						
n (%)	43 (11.9)	8 (2.2)	333 (92.2)	36 (13.6)	10 (3.8)	237 (89.4)
OR (95% CI)*	Reference	**0.4 (0.2 to 0.8)**	**5.9 (3.5 to 9.8)**	Reference	**0.3 (0.1 to 0.6)**	**2.8 (1.5 to 5.0)**
HPV positive, normal						
n (%)	51 (29.3)	36 (20.7)	71 (41.0)	36 (33.3)	24 (22.2)	66 (62.9)
OR (95% CI)*	1.7 (0.9 to 3.1)	Reference	Reference	2.2 (1.3 to 3.7)	Reference	Reference
HPV positive, abnormal						
n (%)	35 (32.7)	22 (20.6)	60 (56.6)	21 (28.8)	17 (23.3)	47 (64.4)
OR (95% CI)*	1.8 (0.9 to 3.6)	0.6 (0.3 to 1.5)	1.8 (1.0 to 3.1)	**1.8 (0.9 to 3.7)**	0.8 (0.4 to 1.7)	1.1 (0.6 to 2.0)
HPV persistent						
n (%)	41 (35.3)	23 (19.8)	61 (52.6)	27 (31.0)	18 (20.7)	54 (62.8)
OR (95% CI)*	**2.00 (1.1 to 3.7)**	0.8 (0.4 to 1.6)	1.5 (0.8 to 2.6)	**2.00 (1.1 to 3.5)**	0.8 (0.4 to 1.5)	1.0 (0.6 to 1.8)

Logistic regression models, fully adjusted for age, marital status, ethnicity, Index of Multiple Deprivation, education, number of previous screens and NHS site.

Bolded ORs are statistically significant.

*OR (95% CI) of having higher worry, higher concern or higher reassurance.

†Includes women not tested for HPV, HPV negative, HPV cleared.

HPV, human papillomavirus; NHS, National Health Service.

### Concern and reassurance related to results

Women in the control group were less likely to be concerned and more likely to feel reassured by their results at 6 and 12 months than those who were HPV positive with normal cytology (table 3). Figure 3 shows the odds of higher concern and higher reassurance for women who were and were not concerned/reassured at baseline. Compared with women who were not concerned about their results at baseline, those who were concerned had higher odds of being concerned at 6-month and 12-month follow-up (OR=8.0, 95% CI: 4.3 to 14.8 and OR=5.0, 95% CI: 2.7 to 9.4, respectively). Similarly, women who were reassured at baseline were more likely to feel reassured at 6 and 12 months (OR=4.5, 95% CI: 3.0 to 6.9 and OR=3.0, 95% CI: 1.8 to 5.1, respectively) compared with women who were not reassured at baseline.

### Receiving an HPV-negative or HPV-positive result at recall

We compared psychological outcomes between those who had attended recall and were HPV negative, were HPV positive and those who had not yet attended (online supplemental table S3). Among women who had been HPV positive with normal cytology at baseline (n=109), those who had reattended and were HPV negative had lower anxiety scores, were less worried, less concerned and more reassured than women who had reattended and were HPV positive or had not yet reattended (p<0.05). There were no significant differences in any of the outcomes by self-reported reattendance/HPV status among women who had been HPV persistent at baseline (n=89, p>0.05).

## Discussion

We have previously shown that testing positive for HPV resulted in higher anxiety and distress than testing negative or not being tested.^11^ This is no longer the case at 12 months and suggests that primary HPV testing is unlikely to have a sustained impact on anxiety or distress. These findings are consistent with a recent systematic review that showed differences in emotional response dissipate over time^13^ and are reassuring given that HPV primary screening has now been implemented in many countries.

The proportion of women who felt concerned about their test result or worried about their risk of cervical cancer reduced over the follow-up period, but remained higher for HPV-positive women. While these specific concerns are not severe enough to raise anxiety, an HPV-positive result may lead to a sense of continued risk of cervical cancer. This could explain high compliance with early recall.^5^ Exploratory analysis of secondary outcomes by self-reported screening result at 12-month recall suggests worry and concern were substantially lower and reassurance higher among women who tested HPV negative.

Women who were HPV positive with abnormal cytology had the highest anxiety and distress at baseline, but scores had fallen almost within the normal range by 6 months, consistent with studies in the triage context.^12^ Although anxiety and distress were slightly higher in our study than for women receiving HPV results alongside cytology,^14^ our findings suggest that for most women with abnormal cytology, the addition of an HPV-positive result has no lasting impact on anxiety or distress.

At 6 months, some women who were HPV positive with normal cytology were still concerned about their test result (21%) and worried about their cancer risk (29%), similar to proportions among women who also had abnormal cytology, despite the difference in clinical risk between these groups. By 12 months, fewer women who were HPV positive with normal cytology felt reassured than HPV-positive women with abnormal cytology, perhaps because those with abnormal cytology would have had follow-up colposcopy and the opportunity to discuss concerns with a clinician.

In our baseline paper,^11^ we found women who had been recalled at 12 months and tested HPV persistent or HPV cleared did not have higher anxiety or distress than the control group, suggesting that it is the initial HPV-positive result that causes raised anxiety. In this paper, there continued to be no differences in these groups for up to 12 months. Overall, women who had persistent HPV at the time of the baseline questionnaire (and had been HPV positive for 24 months by the time of the 12-month questionnaire) remained more worried about cervical cancer. Further research is needed to establish the psychological consequences for women who test HPV positive multiple times.

There are two main strengths of this study. First, it was conducted in a ‘real-life’ context, with participants invited for screening within the NHS programme. Second, we used validated measures of anxiety and distress with recognised clinical cut-offs and women in the control group had State Anxiety Inventory scores close to population norms. Worry, concern and reassurance were assessed using single-item questions as opposed to validated scales, so the clinical significance of these should be interpreted with caution.

The study had a low response rate (22%) and our findings may therefore not be fully generalisable to the population of women invited for screening. While we used weight to adjust for non-response (for age and socioeconomic status), it is likely that those responding were more engaged. This is reflected by higher response rates among HPV-persistent and HPV-cleared women (27%–28%) and lower response rates among those not tested for HPV (16%). It is also possible that collecting data using written questionnaires may have excluded some women, for example, with low literacy. In addition, 90% of our sample were from white (British or other) backgrounds, slightly more than in 25–64 years old in the English population (86%). Response rates for the follow-up questionnaires were good, but only 40% completed the questionnaire at all three time points. There may have been additional biases for the follow-up questionnaires. For example, anxiety levels in our control group were slightly higher at 6 and 12 months than baseline and it is possible that women who felt more anxious were more motivated to return follow-up questionnaires. We were not able to collect clinical data at follow-up (eg, for colposcopy timing/outcomes), so we cannot determine whether women returning follow-up questionnaires represented a particular treatment/outcome.

This work was designed to look at the same outcomes across the year following test results and it may be that other concerns, not assessed, are still present or become present later on. Our data were collected in five sites that were piloting HPV primary screening. Our findings therefore represent psychological responses at the time, before HPV primary screening was fully rolled out in 2019.

## Conclusion

Elevated anxiety and distress following an HPV-positive result in the context of primary HPV screening subside within 6 months and this remains the case until at least 12 months. Concern about developing cervical cancer remains higher at 6 and 12 months among women with an HPV-positive result, supporting the need for early recall to provide reassurance to those no longer at higher risk.

**Key messages**

- Women testing positive for human papillomavirus (HPV) have higher anxiety shortly after receiving their result.
- By 6 months, anxiety scores are similar to women with normal screening results.
- Primary HPV testing is unlikely to have a sustained impact on anxiety or distress.
- Specific concern about results and worry about cancer may be longer lasting, supporting the need for early recall to resolve concern.

## Data Availability

The datasets generated and/or analysed during the study are available from the corresponding author on reasonable request.
