# Supplementary material for: Patterns of anxiety and distress over 12 months following participation in HPV primary screening
Source: Sex Transm Infect. 2021 Aug 3;98(4):255–61. doi: 10.1136/sextrans-2020-054780 (PMC9120391; doi:10.1136/sextrans-2020-054780)
Supplement: Supplementary data [file sextrans-2020-054780supp001.pdf]

Supplementary Figure S1: Longitudinal study flow diagram

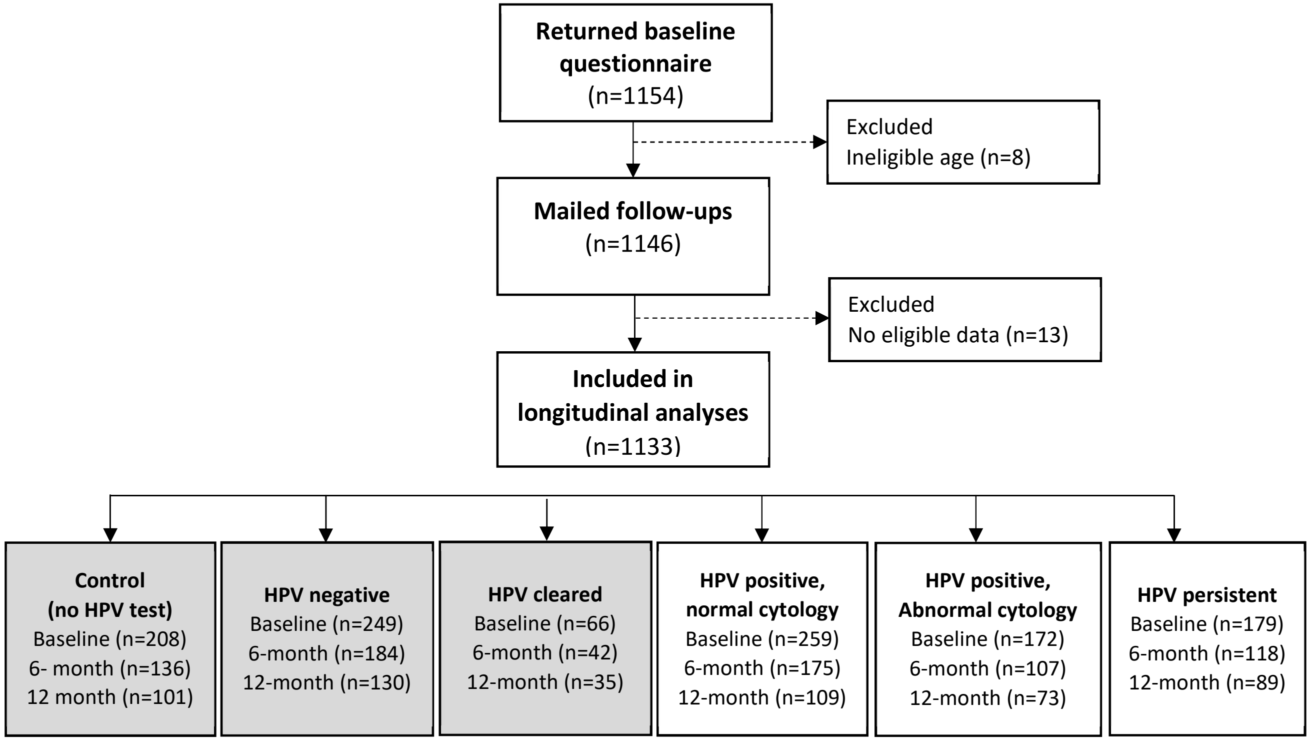

Note 1: Women who were HPV persistent and HPV cleared had tested HPV positive ~1 year earlier and the baseline questionnaire was following their early recall result
Note 2: The overall baseline sample size was slightly greater than reported in McBride et al. (10) due to delayed data entry for six questionnaires.
Note 2: The three groups shaded grey represent the control group for the follow-up analyses

Supplementary Table S1: Attrition scenarios (n=1133)

	n (%)
Data at baseline only	290 (25.6)
Data at baseline and 6-months	306 (27.0)
Data at baseline and 12 months	81 (7.1)
Data at all three time points	456 (40.2)

Supplementary Table S2: Changes in mean anxiety and distress from baseline to 6 and 12 months by result group

	Control	HPV positive, normal	HPV positive, abnormal	HPV persistent
Anxiety [a]				
From baseline to 6 months	Ref	-1.7 (-4.6, 1.3)	**-6.4 (-10.4, -2.4)**	-2.6 (-6.0, 0.7)
From baseline to 12 months	Ref	**-3.6 (-7.0, -0.2)**	**-7.4 (-12.1, -2.8)**	-1.9 (-6.0, 2.1)
Distress [a]				
From baseline to 6 months	Ref	-0.5 (1.3, 0.3)	**-1.0 (-2.0, -0.01)**	-0.3 (-1.2, 0.5)
From baseline to 12 months	Ref	-0.5 (-1.4, 0.3)	**-1.1 (-2.1, -0.09)**	0.1 (-0.9, 1.0)

Fully adjusted for age, marital status, ethnicity, index of multiple deprivation (IMD), education, number of previous screens and NHS site. Weighted by age group and IMD quintile. Multiple imputation used to account for missing data. Bold indicates significance.

Supplementary Table S3: Primary and secondary outcomes by self-reported re-attendance at recall

	**HPV positive, normal (n=109)**				**HPV persistent (n=89)**			
	Re-attended, HPV positive (n=39)	Re-attended, HPV negative (n=31)	Not re-attended/ Not sure of result (n=33)	p-value	Re-attended, HPV positive (n=43)	Re-attended, HPV negative (n=9)	Not re-attended/ Not sure of result (n=35)	p-value
STAI score (mean; SD)	**37.7 (12.7)**	**31.1 (10.7)**	**40.0 (17.0)**	.023	39.5 (12.6)	31.5 (9.8)	34.8 (10.8)	.081
GHQ score (mean; SD)	1.7 (3.1)	2.0 (3.0)	2.9 (3.9)	.345	2.8 (3.2)	2.5 (3.5)	1.9 (3.0)	.425
Higher worry about cancer: n (%)	**21 (53.8)**	**5 (13.2)**	**10 (33.3)**	.001	14 (33.3)	4 (30.8)	9 (28.1)	.891
Higher concern: n (%)	**14 (35.9)**	**4 (10.5)**	**6 (20.0)**	.026	10 (23.8)	2 (15.4)	6 (18.8)	.761
Higher reassurance: n (%)	**20 (52.6)**	**32 (88.9)**	**14 (46.7)**	<.001	27 (64.3)	9 (75.0)	18 (56.3)	.499

Data shown for HPV positive, normal cytology and HPV persistent women only who would have been invited for a recall appointment around the time of the 12-month questionnaire.

Unweighted and unadjusted analyses. Bold indicates significance at p<.05.
